# Dissecting the contribution of EBNA3C domains important for EBV-induced B-cell growth and proliferation

**DOI:** 10.18632/oncotarget.5002

**Published:** 2015-08-18

**Authors:** Hem Chandra Jha, Sanket Kumar Shukla, Jie Lu, Mahadesh Prasad Aj, Shuvomoy Banerjee, Erle S. Robertson

**Affiliations:** ^1^ Department of Microbiology and the Tumor Virology Program, Abramson Comprehensive Cancer Center, Perelman School of Medicine at the University of Pennsylvania, Philadelphia, PA, 19104, United States of America

**Keywords:** EBV, BACmid, MAP kinase, homologous recombination, Nm23-H1

## Abstract

Epstein-Barr virus (EBV) is an oncogenic gammaherpes virus which is linked to pathogenesis of several human lymphatic malignancies. The EBV essential latent antigen EBNA3C is critical for efficient conversion of primary human B-lymphocytes to lymphoblastic cell lines and for continued LCL growth. EBNA3C, an EBV latent antigen with oncogenic potential can bind and regulate the functions of a wide range of cellular transcription factors. In our current reverse genetics study, we deleted the full length EBNA3C, and independently the RBP-Jκ and Nm23-H1 binding sites within EBNA3C using BACmid recombinant engineering methodology. Our experiments demonstrated that deletion of the EBV EBNA3C open reading frame (ORF) and more specifically the residues 621–675 which binds Nm23H1 and SUMO-1 showed a significant reduction in the ability of the cells to proliferate. Furthermore, they exhibited lower infectivity of human peripheral blood mononuclear cells (PBMCs). We also showed that recombinant EBV with deletions of the EBNA3C ORF, as well as a recombinant with residues 621–675 within EBNA3C ORF deleted had diminished abilities to activate CD40. Our study also revealed that the full length (1–992) and 621–675 aa deletions of EBNA3C when compared to wild type EBV infected PBMCs had differential expression patterns for the phosphorylation of MAP kinases specifically p38, JNK and ERK. Regulation of β-catenin also differed among wild type and EBNA3C deleted mutants. These temporal differences in signaling activities of these recombinant viruses in PBMCs is likely important in defining their functional importance in EBV-mediated B-cell transformation.

## INTRODUCTION

Epstein-Barr virus (EBV) is a human gammaherpesvirus that latently infects B-cells. EBV maintains long-term persistence in human B-lymphocytes and causes a number of B-cell malignancies including Hodgkin's lymphoma (HL), Burkitt's lymphoma (BL), and Diffuse large B cell lymphoma (DLBCL) [1]. EBV life cycle has two phases: latent infection and lytic replication and can induce the continuous proliferation of primary peripheral human B-cells infected *in vitro* [2]. The EBV genome comprises about 180 kb of double stranded DNA and encodes at least 86 open reading frames [3]. These genes located in the long unique region of the genome encode nine latent proteins which includes Epstein-Barr nuclear antigen 1 (EBNA1), EBNA2, EBNA3A, -3B, -3C, EBNA-LP, and latent membrane protein 1 (LMP1), LMP2A and -2B, as well as a number of lytic proteins such as the immediate early transactivator BZLF1 (also referred to as ZEBRA or Zta) and the viral polymerase BALF5 [4, 5].

In the field of herpesvirus research, homologous recombination is a widely applied method of genetic engineering for generating mutants [6]. One popular homologous recombination strategy is based on the bacterial artificial chromosome (BAC) system. The BAC system is considered one of the most useful method for molecular cloning of large DNA viruses such as herpesviruses [7] and these recombinant BACmids are important for studying the functions of individual viral genes [8]. Recently, we have generated an EBV-BAC system with a GFP expression cassette for maintaining infection [2]. This recombinant virus successfully infects human peripheral B-cells as seen by a strong GFP signal during early primary infection and also activated CD40 in a time-dependent manner [2]. CD40 is a type I transmembrane glycoprotein belonging to the TNF receptor super family [9] and is expressed on B-cells, follicular dendritic cells, dendritic cells, activated monocytes, macrophages, endothelial cells, vascular smooth muscle cells and several tumor cell lines [9].

Earlier reports suggested that activated CD40 and EBV latent membrane protein 1 (LMP-1) were responsible for EBV reactivation [10]. EBV also activated CD40 signaling and promotes cell survival and proliferation in gastric carcinoma-derived human epithelial cells and in the virus-infected lymphocytes [11]. Importantly, EBV-mediated B-cell proliferation is dependent upon EBV LMP-1, which simulates an activated CD40 receptor [12]. Furthermore, CD40 ligation down-regulates EBNA-2 and LMP-1 expression in EBV-transformed LCLs [13].

The Nm23-H1 protein is a known suppressor of cell migration, tumor metastasis and it is expressed in all tissues. It is widely studied as a potent anti-metastatic factor in human cancers [14]. Enhanced expression of cellular Nm23-H1 is associated with decreased metastasis in breast cancer, melanoma, colon cancer, oral squamous cells, T-cell lymphoma, Hodgkin lymphomas and diffuse large B-cell lymphoma [15–19]. Previous reports also suggested that enhanced expression of Nm23-H1 altered the scaffold properties of KSR1 and inhibited ERK and MAPK signaling [20]. In addition, higher Nm23-H1 expression was found to reduce phosphorylation of ERK in breast cancer cells [21]. Our previous work also demonstrated that proliferation of BJAB cells expressing Nm23-H1 was significantly lower and showed increased apoptotic activities [16]. The EBV essential latent antigen EBNA3C interacted with the human metastatic suppressor Nm23-H1 at sequences located between the glutamine- and proline-rich domains (aa 621–675), and activated transcription [22, 23]. Furthermore EBV modulated the expression of alpha V integrin and the metastasis suppressor Nm23-H1 through interaction with the GATA-1 and Sp1 transcription factors [24]. EBNA3C also modulated the activity of the transcription factor Necdin to regulate expression of its cellular targets to induce metastasis in a nude mouse model [25, 26].

Recombination signal binding protein for immunoglobulin kappa J region (RBP-J) is a major downstream effector of the Notch signaling pathway in mammals which recruits distinct transcription protein complexes to responsive promoters for regulating expression of target genes [27, 28]. The Notch/RBP-Jk complex is involved in stabilization of β-Catenin to promote proliferation as well as suppression of the differentiation process [29]. Interestingly, EBNA3C amino acids 1 to 183 are important for interaction with RBP-Jk [30]. Earlier, it has been shown that residues 180–231 of EBNA3C are critical for EBNA2-induced transcription mediated through RBP-Jκ and deletion of residues 180–231 of EBNA3C was unable to support LCL growth [31].

In this study we generated three EBV recombinants by BAC recombinant engineering, deleted for full-length EBNA3C, residues 621–675 which is the Nm23-H1 binding domain within EBNA3C, and the RBP-Jκ binding site residues 183–240 within EBNA3C, respectively. These recombinant viruses were examined to evaluate the role of EBNA3C, and its binding domains for RBP-Jk and Nm23-H1 in B-cell activation and proliferation during latent and primary infection. Our results demonstrated that deletion of full length EBNA3C, and residues 183–240 and 621–675 within EBNA3C resulted in a significant decrease in proliferation of infected cells. Furthermore, they showed lower infectivity in human PBMCs during the early stages of infection. Interestingly, these two mutants suggest that EBNA3C has a vital role in CD40 activation, MAP kinase activation and β-Catenin stabilization in B-cells infection and transformation.

## RESULT

### Generation of recombinant EBV-GFP viruses deleted for EBNA3C specific residues

**Figure 1:**
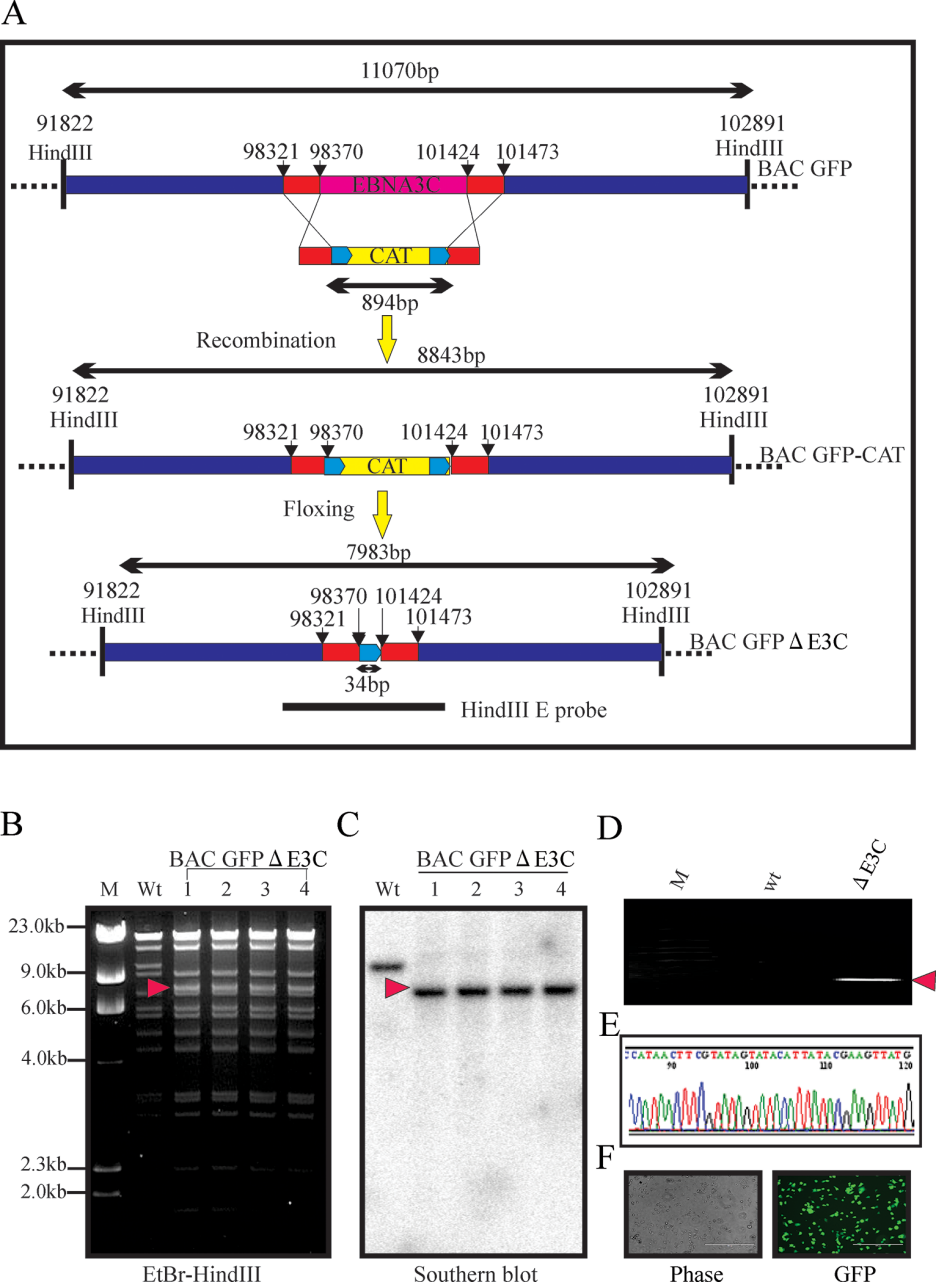
Generation of the recombinant viruses BAC EBV-ΔE3C **A.** Schematic diagram showing generation of BAC EBV-ΔE3C, a recombinant BAC EBV-GFP with deletion of EBNA3C. **B, C.** Ethidium bromide-stained gel and Southern blots with BAC EBV-GFP wt (lane 2) and the mutated BACmid, BAC EBV-ΔE3C, cleaved with HindIII (Lanes 3–6). **D.** PCR analysis for BAC EBV-ΔE3C recombinant virus at the junction of deletion of the EBNA3C ORF. **E.** selected chromatogram of the junction PCR product. **F.** Cells were transfected with BAC EBV-ΔE3C. GFP expression levels were monitored by fluorescent microscopy 2 days after transfection.

Our previous studies showed that EBNA3C contributes to proliferation EBV associated lymphoma [38–44]. The Nm23-H1 and RBP-Jκ binding sites within EBNA3C are located in the carboxy and amino-terminal domains of EBNA3C, respectively. These binding sites were shown to be associated with EBV growth and proliferation [44–46]. However, no further investigations were performed within the viral genome. Here, we constructed three recombinant viruses on the backbone of the BAC-EBV-GFP, a GFP-tagged EBV virus generated previously [2]. The BAC EBV-GFP carries the EBV genome, a GFP tag and resistance genes for ampicillin, kanamycin, and puromycin [2]. Infectious EBV can be produced by transfection of BAC-EBV-GFP into HEK-293T cells, selection followed by chemical induction [2]. Using the BAC EBV-GFP as a template, we designed PCR primers so that the full-length EBNA3C (98,370–101,424 EBV co-ordinates) were removed from the genome. A schematic for the generation of the recombinant BAC GFP-EBV using PCR primers that integrated the chloramphenicol (cat) resistance gene and two loxP sites from plasmid pL452 into the BAC GFP-EBV genome, and replacing the full-length EBNA3C is shown in Figure 1. LoxP is the substrate sequence of Cre recombinase, so the insert fragment between two loxP sites (including CAT cassette) can be subsequently removed by expressing the Cre recombinase after induction by L-arabinose [2]. A PCR product containing the CAT gene flanked by loxP sites and two fragments of 50-bp EBV sequences [47] from the two ends of the full-length EBNA3C was generated using BAC GFP-EBV as a template. This PCR product was transfected into E. coli 350 containing the BAC EBV-GFP to remove full-length EBNA3C after homologous recombination and Cre-mediated excision of the CAT ORF. The resulting BAC recombinants were screened and analyzed on 0.65% agarose and southern blot analysis to show that the full-length EBNA3C was removed from the EBV genome (Figure 1B and 1C). Digestion of the BAC EBV-GFP with HindIII generated a 7,983 bps fragment compared to the 11,0710 bps of the full-length EBNA3C domain suggesting that the full-length EBNA3C was removed at the desired site (Figure 1B and 1C). To further confirm whether the altered digestion pattern of the BAC mutants was the result of the expected recombination, we performed PCR across the junction by using the primers designed at the recombination site. The results showed that the PCR bands in from the EBNA3C ORF shifted based on the presence of the remaining loxP site and HindIII site (Figure 1D). Finally, the PCR products were sequenced to confirm the expected mutation (Figure 1E).

**Figure 2:**
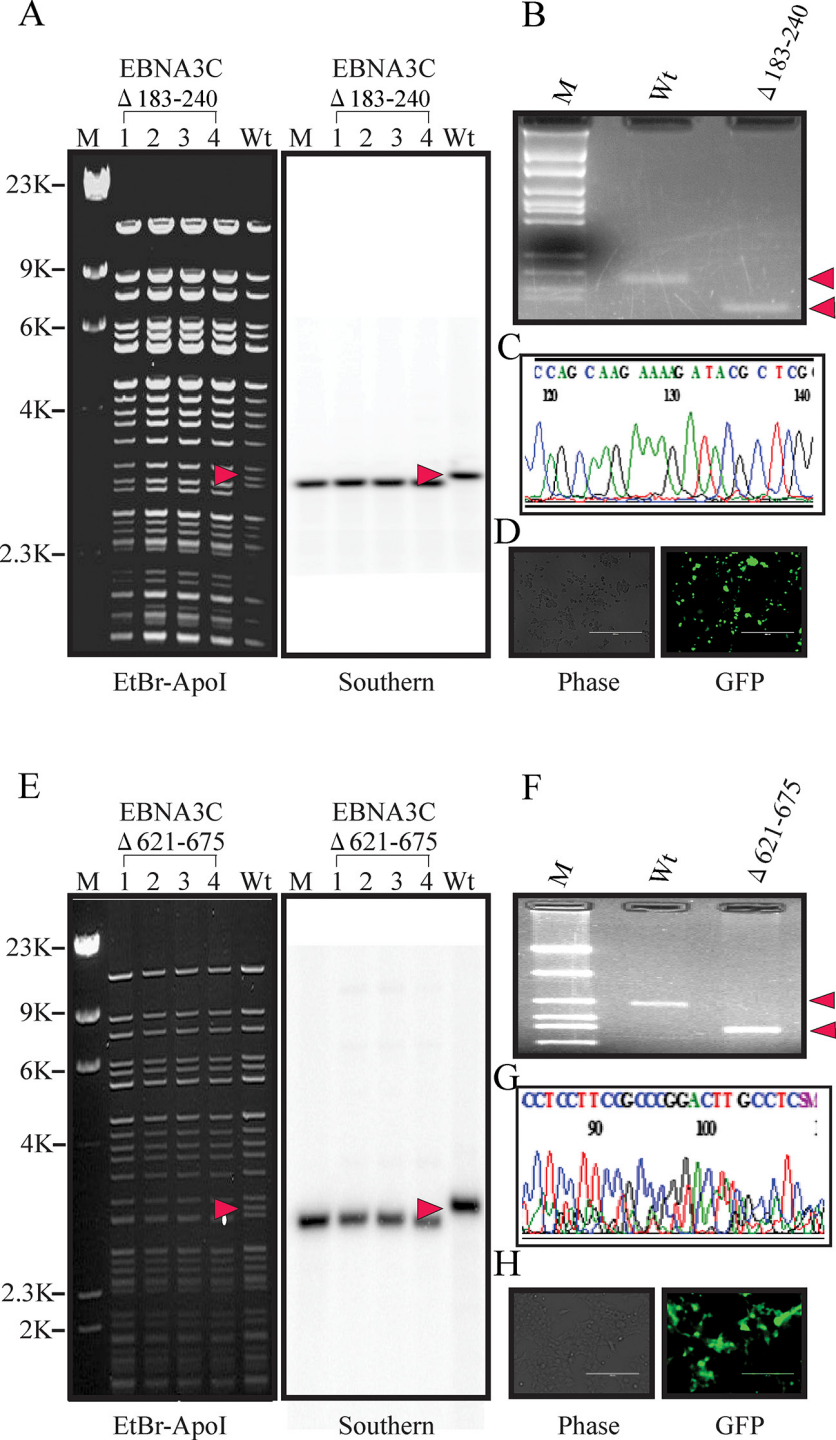
Generation of the recombinant viruses BAC EBV-ΔE3C, 183–240 and EBV-ΔE3C 621–675 **A.** Ethidium bromide-stained gel and Southern blots with BAC EBV-GFP wt (lane 6) and the mutated BACmid, BAC EBV-ΔE3C 183–240, cleaved with ApoI (Lanes 2–5). **B.** PCR analysis for BAC EBV-ΔE3C 183–240 recombinant virus at the junction of the deletion within EBNA3C. **C.** Selected chromatogram of the junction PCR product. **D.** Cells were transfected with BAC EBV-ΔE3C 183–240. GFP expression levels were monitored by fluorescent microscopy 2 days after transfection. **E.** Ethidium bromide-stained gel and Southern blots with BAC EBV-GFP wt (lane 6) and the mutated BACmid, EBV-ΔE3C 621–675, cleaved with ApoI (Lanes 2–5). **F.** PCR analysis for EBV-ΔE3C 621–675 recombinant virus at the junction of deletion of EBNA3C. **G.** Selected chromatogram of the junction PCR product. **H.** Cells were transfected with BAC EBV-ΔE3C 621–675. GFP expression levels were monitored by fluorescent microscopy 2 days after transfection.

For BAC E3CΔ183–240, replacement of the RBP-Jκ binding site with the CAT cassette changed the fragment sizes to 3.2 kb. After induction, the fragment between two loxP sites was removed, producing a fragment size of 3.1 kb- indicating removal of the CAT cassette. Southern blot analysis showed the presence of a 3.2kb band before induction and a unique 3.1kb band in the recombinant EBNA3CΔ183–240 when hybridized with a probe within the RBP-Jκ binding site (Figure 2A right panel). PCR across the new junction generated showed that the bands in the BAC EBNA3CΔ183–240 shifted based on the presence of the remaining loxP site and ApoI site (Figure 2B). Finally, the PCR products were sequenced to confirm the expected mutation (Figure 2C).

Similarly, for E3CΔ621–675, replacement of these residues with the CAT cassette results in a change in fragment size. After induction, the fragment between two loxP sites was removed, so the smaller fragment (3.2 kp) shifted in size to 3.0 kb, indicating removal of CAT cassette. Southern blot showed the presence of a 3.2 kb band before induction and a unique 3.0 kb band in the recombinant BAC EBV-GFP when hybridized with a probe within EBNA3C domain (Figure 2E right panel). To further confirm whether the altered digestion pattern of the BAC mutants was the result of the expected recombination, we performed PCR across the junction by using the primers designed at the recombination site. The results showed that the PCR bands in from the EBNA3C ORF shifted based on the presence of the remaining loxP site and ApoI site (Figure 2F). Finally, the PCR products were sequenced to confirm the expected mutations (Figure 2G).

### Generation of the EBNA3C recombinant viruses

**Figure 3:**
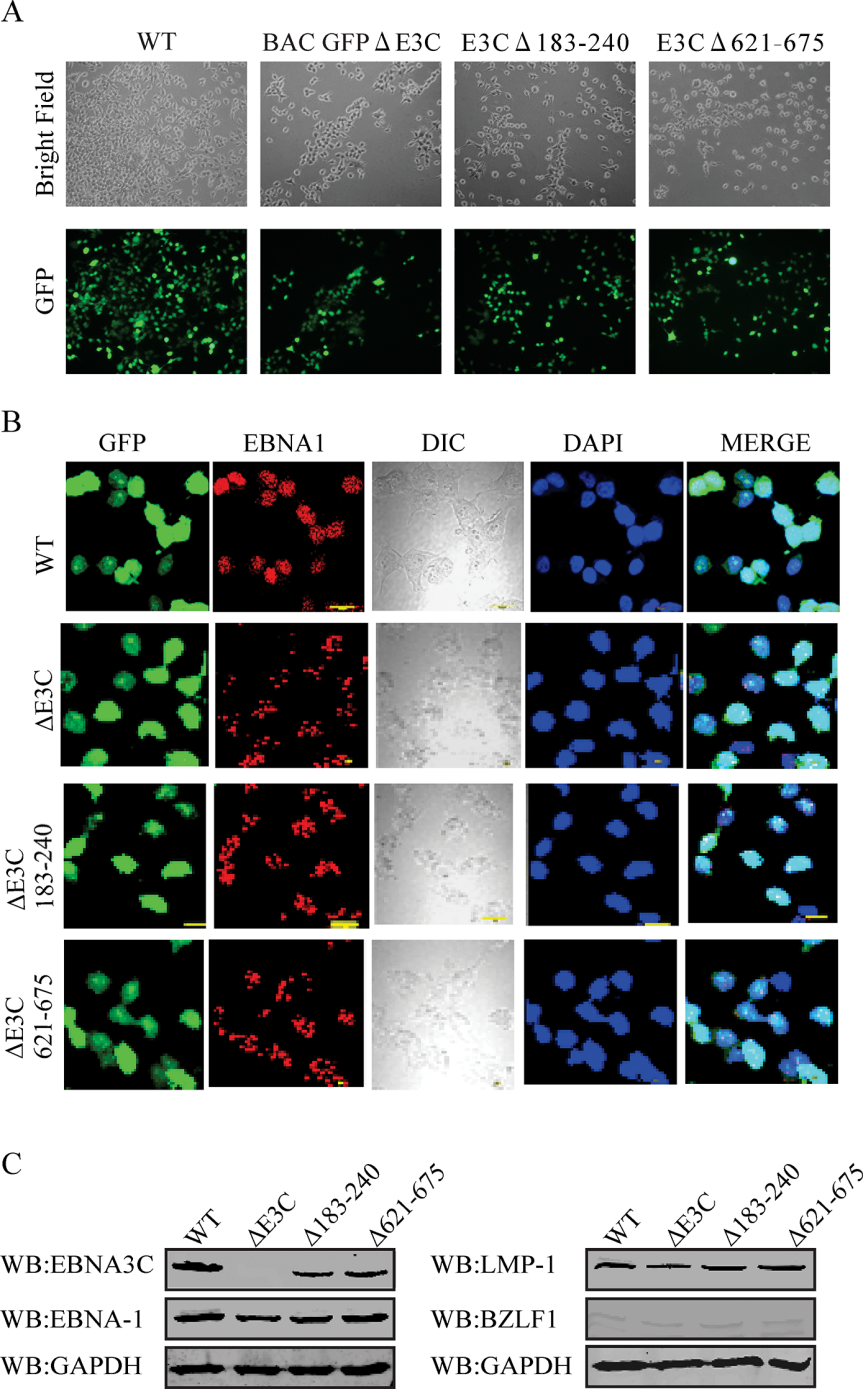
Characterization of BAC EBV-ΔE3C, EBV-ΔE3C 183–240 and EBV-ΔE3C 621–675 stable 293T cell lines **A.** HEK 293T cells were transfected with BAC EBV-ΔE3C, EBV-ΔE3C 183–240 and EBV-ΔE3C 621–675 DNAs. The transfected cells were split and selected with puromycin. The puromycin-resistant cells were pooled and passed for 3–4 weeks. The homogenous population of GFP-positive cells harboring BAC EBV-ΔE3C, EBV-ΔE3C 183–240 and EBV-ΔE3C 621–675 genomes were obtained. GFP expression levels were monitored by fluorescent microscopy **B.** Immunofluorescence analysis for EBNA1 in BAC EBV-ΔE3C, EBV-ΔE3C 183–240 and EBV-ΔE3C 621–675 stable HEK-293T cell lines. **C.** Western blots for EBNA3C, EBNA-1, LMP-1 and BZLF1 for the BAC EBV-ΔE3C, EBV-ΔE3C 183–240 and EBV-ΔE3C 621–675 stable 293T cell lines. GAPDH was an endogenous control.

To reconstitute the recombinant viruses, we transfected EBV GFPΔE3C, EBNA3CΔ183–240 and EBNA3CΔ621–675 into HEK-293T cells. The transfection efficiencies were monitored by fluorescence microscopy for GFP-positive cells which were detected after 24 to 48 hrs of post-transfection (Figure 1F, 2D and 2H). The transfected cells were selected by 1 μg/ml puromycin to generate HEK-293T cell lines harboring EBV GFPΔE3C, EBNA3CΔ183–240 and EBNA3CΔ621–675 DNA (Figure 3A). Subsequently, the cells were fixed and immunostained against EBNA-1, and the results confirmed that EBV GFPΔE3C, EBNA3CΔ183–240 and EBNA3CΔ621–675 stable cell lines expressed EBNA1 and so harbored the EBV genome (Figure 3B). Furthermore, we examined the expression of EBNA3C, EBNA-1, LMP-1 and BZLF1 expression by Western blot analysis. As expected, EBNA3C expression was not detected in EBV GFPΔE3C stable cells. However, EBNA3CΔ183–240 and EBNA3CΔ621–675 showed expression detected at the protein level, though the size was similar or slightly smaller than that seen in the EBV-WT (Figure 3C). In contrast, BZLF1 was hard to detect, suggesting that the stable cell lines are tightly latent with minimal lytic activity (Figure 3C).

### EBNA3C recombinants showed reduced growth activity in colony formation assays

**Figure 4:**
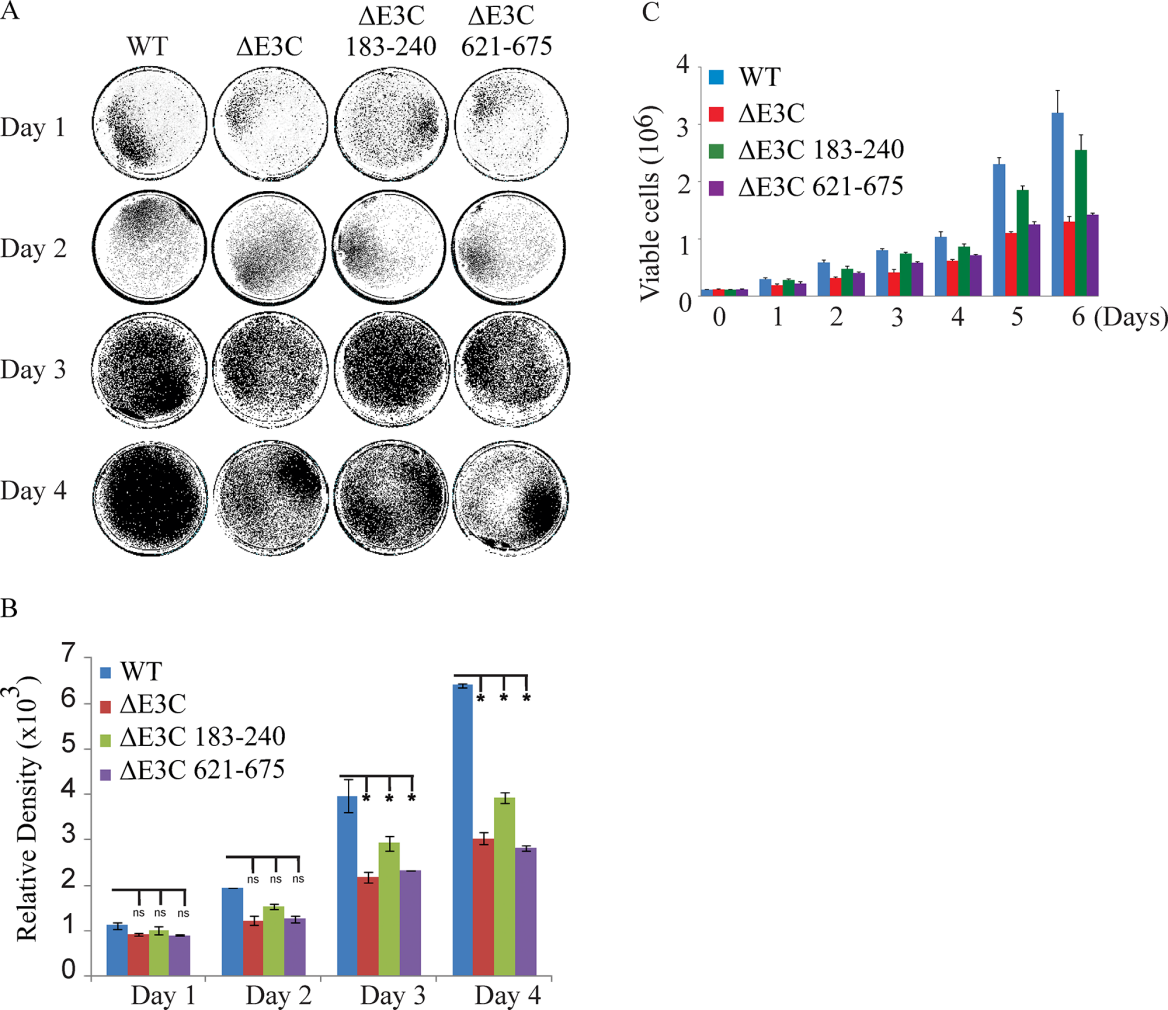
Cell growth assay for BAC EBV-ΔE3C, EBV-ΔE3C 183–240 and EBV-ΔE3C 621–675 stable 293T cell lines **A.** 2 × 10^6^ BAC EBV-ΔE3C, EBV-ΔE3C 183–240 and EBV-ΔE3C 621–675 stable HEK-293T cell lines were plated in DMEM with 5% FBS and cultured in 37°C incubation with 5% CO_2_. The medium were removed and the plates were washed by 1x PBS. The cells were fixed on the plates with 4% formaldehyde and stained with 0.1% crystal violet. The photographs were acquired by Li-Cor Odyssey. **B.** The relative density was quantized using odyssey V 3.0. The number represents the averages of data from three independent experiments. 2-tailed Student's *t*-test was performed to evaluate the significance of differences in the mean values, and *p* values < 0.05 were considered statistically significant and is denoted by an asterisk *. **C.** 1 × 10^5^ million WT-EBV, E3CΔ183–240 EBV, and E3CΔ621–675 EBV expressing HEK-293T cells were subjected to cell proliferation assays by Trypan blue dye exclusion method.

EBV is a ubiquitous human oncovirus and can induce cellular transformation of infected cells [48, 49]. The cells harboring the viral genome have an enhanced capability for driving cell growth. Therefore, to determine the growth rate for EBV GFPΔE3C, EBNA3CΔ183–240 and EBNA3CΔ621–675 infected HEK-293T cells we performed colony formation assays with 0.2 million EBV GFPΔE3C, EBNA3CΔ183–240 and EBNA3CΔ621–675 HEK-293T cells which were seeded in 100 mm petri dish with DMEM plus 5% BGS and 1 μg/ml puromycin. The dishes were collected at 1, 2, 3 and 4 days, washed and fixed on the plates with 4% formaldehyde and stained with 0.1% crystal violet. The colonies were scanned using a LiCor Odyssey scanner and the area of colonies (pixels) in each dish was calculated using the Odyssey V3.0 software. Our results demonstrated in Figure 4A, that the number of colonies for EBV GFPΔE3C HEK-293T cells were decreased 40 ∼ 50% compared to EBV-GFP WT HEK-293T cells at day 3 and 4 (Figure 4). Additionally, the number of colonies for EBNA3CΔ621–675 also showed a 50% decrease when compared to the wild-type EBV-GFP expressing cells at day 3 and 4. Therefore, residues 621–675 which binds Nm23-H1 is important for EBV-mediated cell growth (Figure 4A and 4B). The EBNA3CΔ183–240 infected cells showed an approximately 30% reduction at day 3 and 40% reduction at day 4, suggesting that these residues which contains the RBP-Jκ binding site is also important but to a lesser extent than the residues 621–675 when compared to the EBV GFPΔE3C recombinant (Figure 4B). We further supported our observations by performing cell proliferation assays. Our data demonstrated that cell proliferation was significantly reduced with full length EBNA3C deletion as well as the Δ621–675 recombinant when compared with wild-type EBV infected cells. Similarly, the proliferation rate was not as pronounced with the Δ183–240 recombinant (Figure 4C). The Δ621–675 and the Δ183–240 residues are important in promoting cell growth and proliferation induced by EBV.

### The recombinant viruses EBV GFPΔE3C, EBNA3CΔ183–240 and EBNA3CΔ621–675 can infect human PBMCs

**Figure 5:**
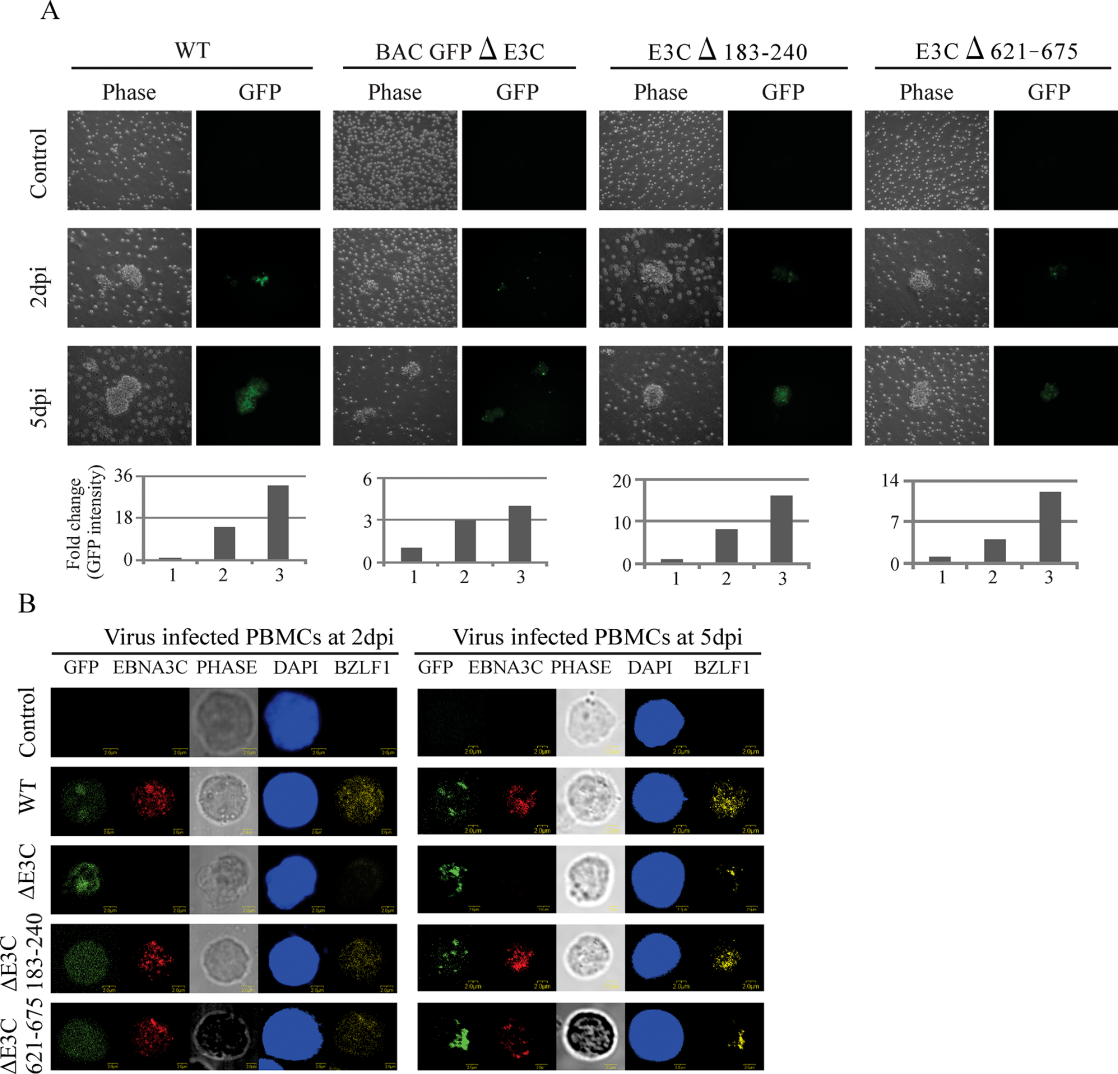
Comparisons of the relative infectivity for BAC EBV-ΔE3C, EBV-ΔE3C 183–240 and EBV-ΔE3C 621–675 recombinant viruses **A.** PBMCs were infected by BAC EBV wt, BAC EBV-ΔE3C, EBV-ΔE3C 183–240 and EBV-ΔE3C 621–675 viruses with equally loading. GFP expression was monitored under a fluorescent microscope after 2, 5 and 7dpi. Intensity of GFP also plotted below the phase and GFP pictures. For quantitation graphs 1, 2 and 3 is represented by controls for day 2 and day 5, respectively. **B.** Immunofluorescence assay for PBMCs infected by BAC EBV-ΔE3C, EBV-ΔE3C 183–240 and EBV-ΔE3C 621–675 recombinant viruses at 2 dpi and 5 dpi. Uninfected and infected PBMCs at 2, and 5 dpi were stained for EBNA3C and BZLF1 protein expression. PBMCs expressed GFP, indicating the presence of viral genome.

Previous studies showed that EBV GFP-WT was highly competent for infecting human PBMCs [2]. Here, we further verified whether these recombinant viruses possess the ability to infect human PBMCs *in vitro*. EBV GFPΔE3C, EBNA3CΔ183–240 and EBNA3CΔ621–675 expressing HEK-293T cells were induced in presence of butyric acid at a final concentration of 3 mM and TPA at a concentration of 20 ng/ml [2]. The supernatant from cell culture were collected and treated with DNAase. The viruses were concentrated by ultracentrifugation 70,000xg at 4°C. The viruses stock were quantified by qRT-PCR. Equal virus particles were added to 1 × 10^6^ PMBCs washed and replaced with complete media. The infected PBMCs were monitored for GFP signal using fluorescence microscopy. Our results showed that EBV GFPΔE3C, EBNA3CΔ183–240 and EBNA3CΔ621–675 infected PMBCs showed detectable signals for GFP at 2dpi and 5dpi (Figure 5A). However, the EBV GFPΔE3C and EBNA3CΔ621–675 recombinants had a weaker signal when compared with EBV GFP-WT and EBNA3CΔ183–240, suggesting that EBV GFPΔE3C and EBNA3CΔ621–675 may have lower infectivity for human PBMCs (Figure 5A). In addition, we performed confocal microcopy staining for specific latent (EBNA3C) and lytic (BZLF1) antibody. To further analyze the infectivity of the recombinant viruses at 3dpi and 5dpi (Figure 5B). The results showed that the EBNA3C signal was clearly present in human PBMCs infected with wt-EBV-BAC-GFP and the Δ183–240 and Δ621–675 EBNA3C recombinants. As expected, no signal was seen for the ΔE3C-GFP-BACEBV (Figure 5B). This shows that the recombinant viruses can infect PBMCs and that the recombinant viruses exhibited some level of lytic replication in these recombinant infected cells similar to wt-EBV-GFP-BAC.

### The EBV GFPΔE3C, EBNA3CΔ183–240 and EBNA3CΔ621–675 recombinant viruses can activate CD19 B-cells

**Figure 6:**
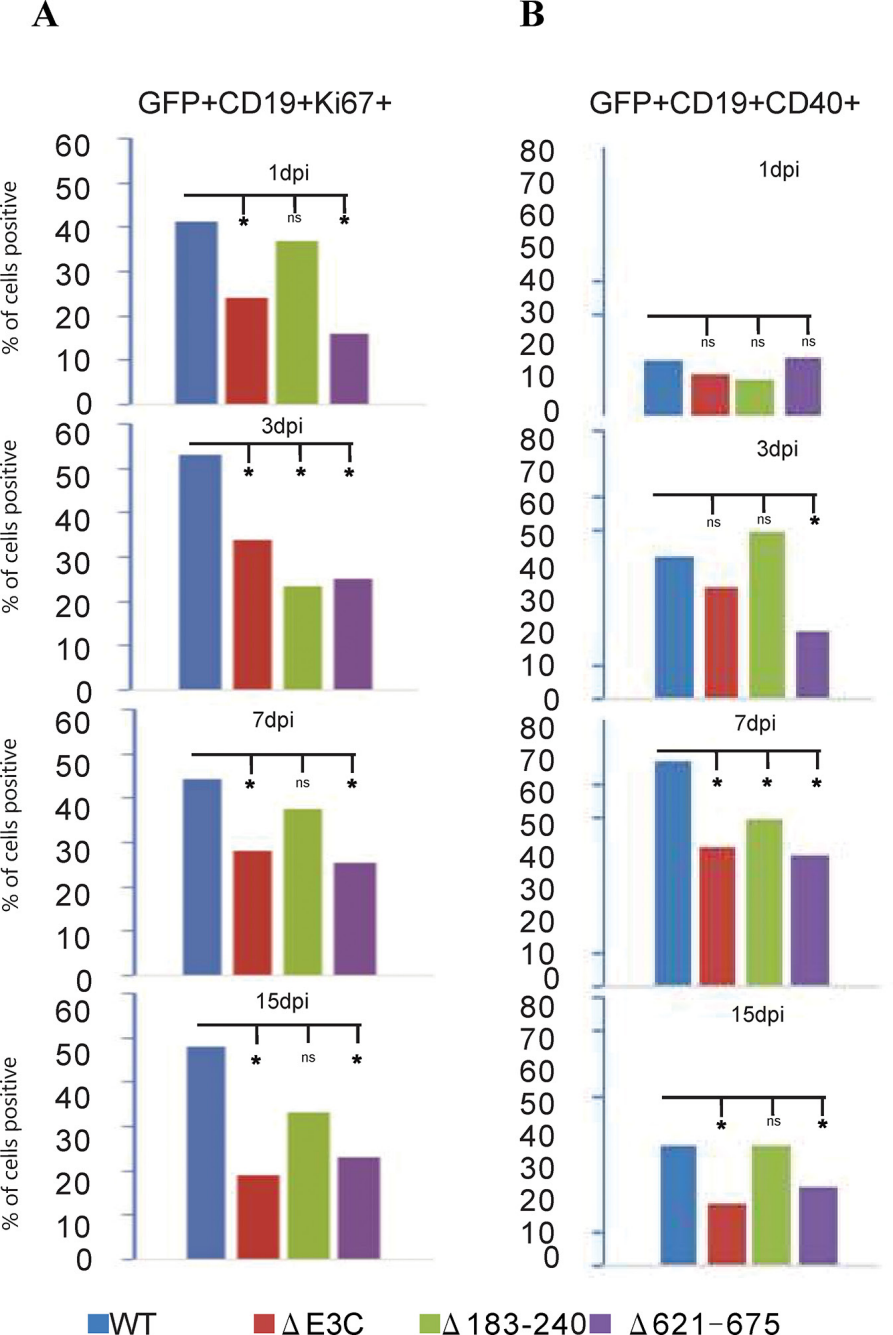
B-cell activation assays of BAC EBV-ΔE3C, EBV-ΔE3C 183–240 and EBV-ΔE3C 621–675 recombinant viruses infected B cells BAC EBV-ΔE3C, EBV-ΔE3C 183–240 and EBV-ΔE3C 621–675 recombinant viruses infected PBMCs were harvested at 1dpi, 3dpi, 7dpi and 15dpi. The B cells were detected by using the PercpCy 5.5 -conjugated anti-CD19 mAbs and PE conjugated anti-Ki67 mAbs were used to monitor the cells proliferation during early infection stage. GFP signals were monitored EBV positive cells. Data were acquired on FACSCalibur equipped with Cell Quest Pro software and analyzed using FlowJo software. 2-tailed Student's *t*-test was performed to evaluate the significance of differences in the mean values, and *p* values < 0.05 were considered statistically significant and is denoted by an asterisk *.

Our previous study demonstrated that EBV infection induces B-cell activation and cell proliferation [2]. Here, we further investigated whether the recombinant viruses are capable of activating B-cells and induce cell proliferation. EBV-GFP-wt, EBV-GFPΔE3C, EBNA3CΔ183–240 and EBNA3CΔ621–675 infected PBMCs were harvested at 1, 3, 7, 15 dpi and stained with PercpCy 5.5 conjugated anti-CD19 mAbs, APC conjugated anti-CD40. These infected human PBMCs were subjected to flow cytometry analysis at the different time points. The expression patterns of the indicated surface antigen markers were determined up to 15 days for the recombinant viruses infected GFP positive cells. Our results showed that the percentage of CD19^+^Ki67^+^ cells with EBV GFP-WT infection was observed higher than those with the mutants infection at 1, 3, 7, and 15 dpi (Figure 6A). Noteworthy, the percentages of CD19^+^Ki67^+^ cells with EBV GFPΔE3C and EBNA3CΔ621–675 infection were found to be substantially lower than those with EBV GFP-WT and EBNA3CΔ183–240 infected CD19 positive B-cells after 24 hours. However, after 3dpi the EBNA3CΔ183–240 and Δ621–675 recombinant infected cells showed levels similar levels of the cell proliferation marker Ki67 which was similar to the EBVΔE3C recombinant which was an approximately 50% drop compared to the EBV-wt recombinant infected cells (Figure 6A). Interestingly, by 15dpi the EBVΔE3C and EBVΔ621–675 recombinants were about 40% of that from the EBV-wt infected cells for Ki67 (Figure 6A). However, the EBVΔ183–240 recombinant was approximately 60% of the EBV-wt infected PBMCs suggesting a slightly greater ability to drive proliferation compared to the EBVΔE3C and EBVΔ621–675 recombinants (Figure 6A). Further studies to look at B-cells activating marker CD40 expressed on infected PBMCs showed that EBV-wt, EBVΔE3C, EBVΔ183–240 and EBVΔ621–675 had similar levels of CD40 activation at 24 hrs post-infection. However, by 15dpi EBVΔE3C and EBVΔ621–675 recombinants showed levels approximately 50% of CD19^+^CD40^+^ cells when compared with EBV GFPΔE3C and EBNA3CΔ621–675 infection which were significantly reduced for CD40 activation (Figure 6B). The EBVΔ183–240 showed similar levels as EBV-wt for CD40 induction of infected PBMCs (Figure 6B).

### EBV recombinants mutated for EBNA3C show reduced activation of the MAPK and Wnt/β-Catenin-mediated signaling pathways

**Figure 7:**
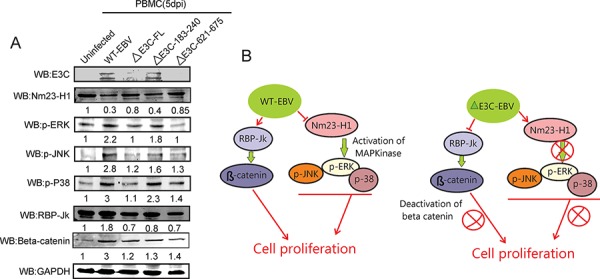
Influence of EBV recombinant mutants in MAPK and Notch/Wnt signaling **A.** Western blot analysis was performed for EBNA3C, p-ERK, pJNK, p-P38 MAPK, RBP-Jk and β-Catenin using appropriate antibodies in the wild type BAC EBV, BAC EBV-ΔE3C, EBV-ΔE3C 183–240 and EBV-ΔE3C 621–675 virus infected PBMCs. GAPDH was taken as internal loading control. **B.** The schematic shows the vital role of EBNA3C and Nm23-H1 binding site within EBNA3C for modulating Nm23-H1 mediated MAPK signaling. Interestingly deletion of the RBP-Jκ site did not have as great as effect on Wnt/β-Catenin pathways.

An earlier report suggested that the ERK MAPK signaling pathway is activated by enhanced expression of Nm23-H1 [20]. It was also reported that Nm23-H1 inhibits the Ras/MAPK pathways which are involved in cancer invasion [50]. Here, we examined the phosphorylation status of the MAPK signaling molecules p-ERK, p-JNK, and p-P38 in EBV-GFP-wt, EBV GFPΔE3C, EBNA3CΔ183–240, EBNA3CΔ621–675 virus infected primary cells. Our results showed reduction in Nm23-H1 expression levels on EBV infection and a substantial induction in ERK, P38 and JNK phosphorylation (Figure 7A & B). However, in the EBVΔE3C, and EBV EBNA3CΔ621–675 recombinant infected cells when compared to wild type virus and EBNA3CΔ183–240 little or no change was seen compared to uninfected (Figure 7A). Previous reports also showed that the Notch/RBP-Jk complex is important for stabilization of β-Catenin which not only promotes cellular proliferation but also impedes the differentiation process [29]. Interestingly, Western blot analysis with recombinant EBVΔE3C, and EBV EBNA3CΔ183–240 infected cells demonstrated an approximately 50% reduction in RBP-Jk protein levels compared to wt control. A 300% induction in β-Catenin protein levels was observed for wtEBV infection and increased level in the EBNA3C recombinants (Figure 7A). These results suggests an important role for EBNA3C and the residues Δ621–675 shown to be important for binding Nm23-H1 in activation of the MAPK signaling pathways (Figure 7B). The deletion of residues 183–240 which contains the RBP-Jk binding site had little or no obvious effect on Wnt/β-Catenin signaling as determined from β-catenin levels (Figure 7A & B). This differed substantially from the EBV ΔEBNA3C deleted virus and the Δ621–675 recombinant virus which showed a 10–20% increase in β-catenin signal.

## DISCUSSION

EBNA3C, one of the Epstein-Barr virus (EBV)-encoded essential latent antigen, is necessary for primary B-cell transformation [51]. EBNA3C was shown to interact with a wide range of transcription factors and modifiers, such as p300 [38], Cyclin A, RBP-Jκ, c-Myc [42], Nm23-H1 [39], SUMO1/3 [52], HDAC1 [53], CtBP [54], DP103 [55], Prothymosin-α [56], p53 [57] and its regulatory proteins Mdm2, ING4, ING5 and Aurora kinase B [44–46]. Earlier reports demonstrated that deletion of EBNA3C has a significant effect on lymphoblastic cell growth [58]. In our study, we observed that deletion of EBNA3C had a substantial effect on growth and proliferation of recombinant virus infected cells. Furthermore, EBNA3C deleted recombinant viruses had lower infectivity of human primary B-cells which were also reduced for B-cell growth and proliferation. This data supports a crucial role for EBNA3C in lymphoblastic cell growth and proliferation during early stages of infection.

Previous reports suggested that EBNA3C can directly interact with RBP-Jκ *in vitro* [59]. EBNA3C lacking residues 180–231, which mediate RBP-Jk association and are necessary for EBNA3C-mediated abrogation of EBNA2-induced transcription through RBP-Jk, also was unable to support growth of LCLs [31]. EBNA3C contains a small “WTP” sequence. This EBNA3C WTP motif can bind RBP-Jk *in vitro*, in yeast, and in mammalian cells [60]. Furthermore, an EBNA3C mutation (W227S) impaired beta trefoil domains binding whereas EBNA3 homology domain mutations disrupted RBP-Jk N-terminal domain binding. However, WTP was not essential for EBNA3C repression of EBNA2 in reporter assays or for maintenance of LCLs growth [60]. Interestingly, promoters which contain RBP-Jκ sites are likely to be differentially regulated by complexes of RBP-Jκ and EBNA3C [61]. Therefore, a deletion of EBNA3C residues at 180–231 are necessary for EBNA3C to inhibit EBNA2-induced transcription through RBP-Jκ, and reduction of LCLs growth *in vitro* [31]. However, our studies showed that deletion of this domain had less of an effect on proliferation of epithelial cells as well as B-cell activation after infection of human PBMCs. Our results therefore indicated that EBNA3C encoded by EBV possesses complimentary mechanisms involved in regulation of B-cell activation and proliferation.

Nm23-H1 is the well-studied anti-metastatic factor associated with human cancers [14]. Nm23-H1 plays a crucial role in limiting tumor cell motility and progression induced by several tumor viruses such as Kaposi's sarcoma associated herpes virus (KSHV) [62], human papilloma virus (HPV) [63], and EBV [14]. For example, KSHV-encoded latency-associated nuclear antigen (LANA) increases expression and nuclear translocation of Nm23-H1, and then activates Ras-B-Raf-MAPK pathway and suppresses KSHV-induced invasiveness [62]. Also, HPV-16 E7 oncoprotein can interact with Nm23-H1 and promotes cell transformation and tumor progression [63]. EBNA3C was shown to interact with Nm23-H1 regulating transcription, cell transformation and cell migration in EBV induced cancers [43]. Studies also demonstrated that the EBV encoded EBNA1 antigen upregulates Nm23-H1 expression and contributes to induced metastasis of nasopharyngeal carcinoma [64]. In this report, a recombinant EBV EBNA3CΔ621–675 clearly showed reduced infectivity in human B-cells. Undoubtedly, this domain which binds Nm23-H1 exhibited an ability to control cell growth and proliferation further suggesting that EBNA3C-mediated deregulation of Nm23-H1 plays an important role in EBV-induced B-cell lymphoma.

The normal resting B-cells infected with EBV results in activation to lymphoblastoid phenotype that are similar to those generated by physiological stimulation with CD40L/IL4 [65]. EBV-encoded LMP1 mimics CD40 signals in B-cells and controls viral activation [10, 66]. CD40 signals are clearly important to the survival and proliferation of virus-infected lymphocytes and EBV-infected epithelial cells [11]. Our results also showed that an EBNA3C deficiency substantially decreases the CD40 activation in B-cells, indicating that EBNA3C has a role in driving EBV infected B-cells during early infection. Furthermore, deletion of a region important for Nm23-H1 binding within EBNA3C has a similar effect to deletion of the full length EBNA3C on CD40 activation of B-cells. Therefore Nm23-H1 binding plays a major role in EBV-mediated CD40 activation of B cells-during early infection.

In summary, EBV encoded EBNA3C is essential for efficient conversion of primary human B-lymphocytes to LCLs and continued growth of the infected LCLs [31]. Here, we deleted the full length EBNA3C, RBP-Jκ and Nm23-H1 binding sites within EBNA3C using BACmid recombinant engineering. The growth assays demonstrated that deletion of EBV EBNA3C and these two domains within EBNA3C showed a reduction in these recombinants infected cells to drive cell proliferation. These recombinants also exhibited a lower infectivity in human PBMCs. Further investigation revealed that EBNA3C through its interaction with RBP-Jk and Nm23-H1 are important effectors of CD40 activation of EBV-infected B-cells. Interestingly, deletion of the Nm23-H1 binding site within EBNA3C had a dramatic effect on its ability to activate CD40 on infected B-cells early during infection. Therefore EBNA3C, and its association with RBP-Jk and Nm23-H1 contribute to growth and activation of B-cells. We also explored the regulation of MAPK and Wnt/β-Catenin signaling pathways in these recombinant virus infected cells. Our results suggest a contributory role of Nm23-H1 and its interaction with EBNA3C to induce MAPK signaling important for B-cell transformation (Figure 7B). This study provides new insights into a potential new strategy for EBV-mediated targeting of the cellular metastatic suppressor Nm23-H1 and further supports a role for RBP-Jk in the context of EBV pathogenesis.

## MATERIALS AND METHODS

### Cells and antibodies

Wild type and mutant viruses expressing HEK-293T cells were cultured in DMEM with 5% bovine growth serum (Gibco, Carlsbad, CA). De-identified PBMCs were provided from the Human Immunology Core at the University of Pennsylvania. The core maintains an IRB approved protocol in which the declaration of Helsinki protocols was followed and each donor gave written informed consent. PBMCs were maintained in RPMI with 10% fetal bovine serum (FBS) (Hyclone, South Logan, Utah). A10 (EBNA3C), and S12 (LMP1) hybridomas were described previously [32, 33]. BZLF1 antibody was provided by Martin Rowe (University of Birmingham, UK) [34]. EBNA1 antibody was purchased from Advanced Biotechnologies, Inc., Columbia, MD. β-Catenin antibody was procured from Cell Signaling, Inc. Beverly, MA. Rabbit polyclonal RBP-Jk antibody was provided by Elliott Kieff (Harvard Medical School, Boston, MA). Nm23-H1 antibody was procured from Seikagaku Corp (Tokyo, Japan). The p-ERK (E-4), p-JNK (G-7), p-P38 (D-8) antibodies were purchased from Santa Cruz Biotechnology, Inc (Santa Cruz, CA), and the GAPDH antibody was obtained from US-Biological Corp. (Swampscott, MA).

### Constructs of BAC EBV-GFP recombinants

BAC EBV-GFP were generated as described previously [2]. Mutagenesis of BAC EBV-GFP was performed using lambda red-mediated homologous recombination engineering [35]. The CAT cassettes were amplified by PCR to include a Hind III site. The primers used for generating the EBV recombinants are listed in Supplementary Table S1.

### Induction and purification of recombinant viruses

Constructs of BAC EBV-GFP mutations were transfected into HEK-293T cells. After 2 days incubation, cells were treated by 0.05% trypsin and plated in 12-well plate in DMEM medium with 5% BGS. 24 hrs later, the medium was replaced with fresh DMEM with 1 μg/ml puromycin every 2 days until the cells were selected to stable cells harboring EBV episomes. The stable cell lines were confirmed by visualization of GFP protein by immunoflorescence. 20 ng/ml TPA (Sigma-Aldrich, St. Louis, Missouri) and 3 mM butyric acid was used for lytic induction [2]. Cell suspensions were centrifuged at 3000 rpm for 20 min and the supernatant was filtered through a 0.45 mm cellulose acetate filter. The viral particles were concentrated by ultracentrifugation at 23,500 rpm at 4°C and stored at −80°C.

### Colony formation and cell proliferation assays

BAC EBV-wt, BAC EBV-ΔE3C, BAC EBV-183–240, and BAC EBV-621–675 were transfected into HEK-293T cells. The cells were monitored and selected to generate stable cell lines with puromycin. 10,000 cells per samples were seeded in 12 cm Petri dish in DMEM supplemented with 5% BGS. After 1, 2, 3 and 5 days, the cells were fixed on the plates with 3% formaldehyde and stained with 0.1% crystal violet. The amount of the colonies in each dish was scanned by an Odyssey scanner (LI-COR Biosciences, Lincoln, NE) and the colony number was quantitated using the Odyssey V3.0 software. 1 × 10^5^ WT-EBV, ΔE3C EBV, E3CΔ183–240 EBV, and E3CΔ621–675 EBV stable HEK-293T cells were plated in 6-well plates and grown in DMEM medium for 6 days at 37°C incubation. Viable cells were counted daily using an automated cell counter by Trypan blue dye exclusion technique.

### Immunoblotting and immunoflorescence assays

Cells were lysed with RIPA buffer (10 mM Tris, pH 7.5, 1% Nonidet-P40, 2 mM Na_2_EDTA, 150 mM NaCl plus protease inhibitors) and protein concentration determined by Bradford assay. The lysates were analyzed by Western blots using antigen specific primary antibodies and infra-Red-tagged secondary antibodies. The results were scanned with an Odyssey Infrared scanner. Densitometry analysis was performed using the Odyssey V3.0 software. Cells were collected and fixed to the slides with 4% paraformaldehyde with 0.1% Triton X-100 for 20 min, and subsequently blocked with 10% BSA at room temperature for 30 min. Cells were incubated with primary antibody (Mouse anti-EBNA1 or EBNA3C, Rabbit anti-BZLF1), and specific signals were detected with secondary antibody conjugated with Alexa Fluor 488, 594 or 647 (Invitrogen, Carlsbad, CA). The cells were counterstained with 4′, 6′-diamidino-2-phenylindole (DAPI). The results were visualized with a Fluoview FV300 microscope (Olympus Inc., Melville, NY).

### Flow cytometry assays

Infected human PBMCs were stained essentially as described previously [2]. Briefly, PBMCs were collected and washed twice with PBS, containing 0.1% BSA and 0.0001% NaN3 at 1, 2, 4 and 7 days post infection. The samples were stained with PercpCy 5.5 conjugated anti-CD19 mAbs, APC conjugated anti-CD40 mAbs and PE conjugated anti-Ki67 mAbs (BD Biosciences, San Jose, CA). GFP signals were used to monitor EBV positive cells. Data were acquired on FACSCalibur equipped with Cell Quest Pro software and analyzed using FlowJo software (Tree Star Inc., Ashland, OR).

### RT-PCR

Total RNAs and cDNAs were prepared as described previously [36, 37]. RT-qPCR was performed on a StepOnePlus Real-Time PCR System (Applied Biosystems Inc, Carlsbad, CA). The reactions were carried out in a 96-well plate at 95°C for 10 min, followed by 35 cycles at 95°C for 20s, 52°C for 30s and then 72°C for 30s. The differences of cycle threshold values (CT) between the samples (ΔCT) were calculated after standardization by GAPDH and converted to fold changes using one of the samples as a standard (1-fold). The primers used were EBNA-1: 5′ CACCATTGAGTCGTCTCCCC 3′, 5′ TCAAAGCTGCACACAGTCAC 3′; EBNA3C: 5′ AGAAGGGGAGCGTGTGTTGTG 3′, 5′ ACGGCA GGAGGCCCAGTATC 3′; GAPDH 5′ GGTCTACAT GGCAACTGTGA 3′, 5′ ACGACCACTTTGTCAAGCTC 3′. All reactions were performed in triplicates.

### Statistical analysis

The data presented here are mean values with standard errors of the means (SEM). 2-tailed Student's *t*-test was performed to evaluate the significance of differences in the mean values, and *p* values < 0.05 were considered statistically significant.

## SUPPLEMENTARY TABLE


