# Supplementary material for: Dissecting the contribution of EBNA3C domains important for EBV-induced B-cell growth and proliferation
Source: Oncotarget. 2015 Aug 18;6(30):30115–29. doi: 10.18632/oncotarget.5002 (PMC4745785; doi:10.18632/oncotarget.5002)
Supplement: Supplementary file 1 [file oncotarget-06-30115-s001.pdf]

SUPPLEMENTARY TABLE

Supplementary Table S1: primer list

EBV delta E3C

Sense:
5′ CAATTTTAGATGAGGTAGAAATTTTGCATATTTTC<br>AGACCCACCATAACTTCGTATAGTATACATTATACGAAGTTATAAATCCTGGTGTCCCTGTTG 3′
Antisense:
TGTCAGTCAAAGATCAAATAGCTAACAGGGGTCACCTTGGATCCCC<br>ATAACTTCGTATAATGTATACTATACGAAGTTATTTACGCCCCGCCCTGCCACT 3′

EBV delta E3C 183-240 [LoxP sites –Red; CAT seq-Green]

Sense:
5′ACATTGGCTTCTAACATCTCCCAGCCAATCCTGGCCCATGGG<br>ATAACTTCGTATAGTATACATTATACGAAGTTATAAATCCTGGTGTCCCTGTTG 3′
Antisense:
5′ACCGCCTCTGCCATTTACCACGAAGGAAGCGTACCTCGGCTTCCCG<br>ATAACTTCGTATAATGTATACTATACGAAGTTATTTACGCCCCGCCCTGCCACT 3′

EBV delta E3C 621-675 [LoxP sites –Red; CAT seq-Green]

Sense:
5′GCCGCGGGGCCTCACATAGTGACGCCTCCTTCCGCCCGGCCTAATCCTGGCCCATGGG<br>ATAACTTCGTATAGTATACATTATACGAAGTTATAAATCCTGGTGTCCCTGTTG 3′
Antisense:
CCGAGAGGGCGCAGACTGATGGGGCCCATGATGGGCGAGGCGTTGT<br>ATAACTTCGTATAATGTATACTATACGAAGTTATTTACGCCCCGCCCTGCCACT 3′
GFP LoXp antisense
AGCGCAACGCAATTAATGTGAGTTAGCTCACTCATTAGGCACCCCAGGCTATAACTTCGTATAATGT
GFP LOXP SENSE
TATCCGCTCACAATTCCACACAACATACGAGCCGGAAGCATAAAGTGTAAATAACTTCGTATAGTATACATTATA
These primers are utilized for inserting GFP to EBV-BACmid.
E3C PR2
GATGTGGTGCCCTGGGATCC
E3C PF2
CGTGAGTCCTTCGGATACTGGG
E3C PF4
TCCCACCGTGAGTCCTTCG
E3C PR4
GGGGTTGTTCTTCGTGCGA
E3C PF3
CGTGTGACCCATGTTTCCATTAA
E3C PR3

(*Continued*)

**EBV delta E3C 621-675 [LoxP sites –Red; CAT seq-Green]**
GGCTTAAAGTTTCCGATTGTCTTG
CAT GENE SENSE
ATAACTTCGTATAGTATACATTATACGAAGTTATAAATCCTGGTGTCCCTGTTG
CAT GENE ANTISENSE
ATAACTTCGTATAATGTATACTATACGAAGTTATTTACGCCCCGCCCTGCCACT
